# Motor-kognitives Risikosyndrom – Weichenstellung für die Demenzprävention?

**DOI:** 10.1007/s00115-023-01487-3

**Published:** 2023-05-04

**Authors:** Paula Theobald, Fabian Herold, Thomas Gronwald, Notger G. Müller

**Affiliations:** 1 Professur für degenerative und chronische Erkrankungen, Bewegung, Fakultät für Gesundheitswissenschaften Brandenburg, Universität Potsdam, Am Mühlenberg 9, 14476 Potsdam, Deutschland; 2 Professur für Sportwissenschaften, Institute of Interdisciplinary Exercise Science and Sports Medicine, MSH Medical School Hamburg, Am Kaiserkai 1, 20457 Hamburg, Deutschland

**Keywords:** Demografischer Wandel, MCI, Risikofaktoren, Kognition, Gang, Demographic change, MCI, Risk factors, Cognition, Gait

## Abstract

**Hintergrund:**

Der prognostizierte starke Anstieg demenzieller Erkrankungen wird auch das deutsche Gesundheitssystem vor große Herausforderungen stellen. In diesem Zusammenhang haben präventive Maßnahmen bei Personen mit einem erhöhten Risiko für eine spätere Demenz eine herausragende Bedeutung. In der englischsprachigen Literatur hat sich diesbezüglich das Konzept des motor-kognitiven Risikosyndroms (MCR) etabliert, welches in Deutschland bisher noch kaum Verbreitung gefunden hat.

**Fragestellung:**

Was sind die Charakteristika und Diagnostikkriterien des MCR? Welche Auswirkung hat das MCR auf gesundheitsrelevante Parameter? Welche Evidenz liegt hinsichtlich der Risikofaktoren und der Prävention des MCR vor?

**Material und Methode:**

Die englischsprachige Literatur wurde in Bezug auf das MCR, dessen Risiko- und Schutzfaktoren sowie auf Zusammenhänge mit der leichten kognitiven Störung (MCI) und dem zentralen Nervensystem analysiert.

**Ergebnisse:**

Das MCR ist durch eine subjektive kognitive Beeinträchtigung und eine verlangsamte Ganggeschwindigkeit gekennzeichnet. Im Vergleich zu gesunden älteren Erwachsenen weisen Erwachsene mit dem MCR neben einem erhöhten Demenzrisiko auch ein erhöhtes Sturz- und Mortalitätsrisiko auf. Modifizierbare Risikofaktoren bieten einen Anknüpfungspunkt für gezielte lebensstilbezogene Präventionsmaßnahmen.

**Schlussfolgerung:**

Aufgrund der praxisorientierten Diagnostik könnte sich das MCR auch im deutschsprachigen Raum als ein wichtiges Konzept zur Früherkennung von Personen mit einem erhöhten Demenzrisiko erweisen, wenngleich weitere Forschung notwendig ist, um diese Annahme empirisch abzusichern.

## Abstract (en)

**Background:**

The predicted increase in adults with dementia will pose a major challenge for the German healthcare system. To mitigate this challenge, the early detection of adults with an increased risk of dementia is crucial. In this context, the concept of motoric cognitive risk (MCR) syndrome has been introduced into the English literature but is currently relatively unknown in German-speaking countries.

**Objective:**

What are the characteristics and diagnostic criteria of MCR? What is the impact of MCR on health-related parameters? What is the current state of evidence regarding the risk factors and prevention of the MCR?

**Material and methods:**

We reviewed the English language literature concerning MCR, the associated risk factors, and protective factors, similarities or differences with the concept of mild cognitive impairment (MCI), and its influence on the central nervous system.

**Results:**

The MCR syndrome is characterized by subjective cognitive impairment and a slower gait speed. Compared to healthy adults, adults with the MCR have a higher risk of dementia, falls, and mortality. Modifiable risk factors provide a starting point for specific multimodal lifestyle-related preventive interventions.

**Conclusion:**

As MCR can be easily diagnosed in practical settings, it could become an important concept for the early detection of adults with an increased risk of dementia in German-speaking countries, although further research is necessary to empirically confirm this assumption.

Aufgrund des demografischen Wandels und der steigenden Lebenserwartung wird ein drastischer Anstieg demenzieller Erkrankungen prognostiziert [30], was zu großen Herausforderungen infolge der pflegeaufwendigen und kostenintensiven Versorgung von Betroffenen führt [24]. Bedingt durch das derzeitige Fehlen eines kausalen und effektiven pharmakologischen Therapieansatzes zur Behandlung demenzieller Erkrankungen [28] sind insbesondere eine frühzeitige Diagnostik und die rechtzeitige Durchführung präventiver Interventionsmaßnahmen wichtige Elemente, um diesen Herausforderungen zu begegnen [20].

Im internationalen und auch im deutschsprachigen Raum hat sich das Konzept der leichten kognitiven Störung (engl.: „mild cognitive impairment“, MCI) etabliert, welches ein Stadium zwischen dem normalen kognitiven Alterungsprozess und einer manifesten Demenz beschreibt [18]. Die Diagnose einer MCI (Tab. 1) kann allerdings aufgrund der durchzuführenden ausführlichen neuropsychologischen Testung zeitaufwendig sein und setzt besonders geschultes Personal voraus [15, 18]. Häufig verwendete Kurztests, wie der Mini-Mental Status Test (MMSE) und das Montreal Cognitive Assessment (MoCA) sollten aufgrund der zu geringen Sensitivität der Testungen für MCI nicht als alleiniges Diagnoseinstrumente verwendet werden [15]. Eine praxisnahe Implementierung des MCI in ressourcenlimitierten Settings (z. B. im Klinikalltag mit limitieren Zeit- und Personalressourcen) ist daher schwierig und erklärt möglicherweise, weshalb diesem Konzept im Klinikalltag teilweise ein untergeordneter Stellenwert zukommt [15].

**Table 1:** 

	MCI	MCR
*Diagnostik*	(i) Abfrage subjektiver kognitiver Einschränkungen (ii) Bestehende Alltagsaktivitäten (iii) Fehlen einer Demenz **(iv) Erfassung objektiver kognitiver Einschränkungen mittels neuropsychologischer Assessments**	(i) Abfrage subjektiver kognitiver Einschränkungen (ii) Bestehende Alltagsaktivitäten (iii) Fehlen einer Demenz **(iv) Untersuchung der Ganggeschwindigkeit**
*Prävalenz*	**19,3** **%**	**10** **%**
*Konversionsrate zu Demenz*	**HR: 3,3**	**HR: 1,38–3,27**
*Bisher bekannte modifizierbare Risikofaktoren*	Geringer Bildungsstatus Depressionen Kardiovaskuläre Erkrankungen Adipositas Diabetes Rauchen Körperliche Inaktivität Schlechte Ernährung **Erhöhte Cholesterinwerte**	Geringer Bildungsstatus Depressionen Kardiovaskuläre Erkrankungen Adipositas Diabetes Rauchen Körperliche Inaktivität Schlechte Ernährung Multiple Stürze Schmerzen Polypharmazie **Erhöhter BMI**
*Bisher bekannte nichtmodifizierbare Risikofaktoren*	Alter	Alter

*BMI* Body-Mass-Index, *HR* Hazard Ratio, *MCI*  mild cognitive impairment , *MCR* motor-kognitives Risikosyndrom

^a^Unterschiede zwischen den Konzepten sind „fett“ hervorgehoben

Eine praxisnähere Alternative und Ergänzung zum Konzept des MCI stellt das im Jahr 2013 eingeführte Konzept des motor-kognitiven Risikosyndroms (engl.: „motoric cognitve risk syndrome“, MCR) dar, welches erstmals von Verghese und Kolleg:innen beschrieben wurde [35]. In der deutschen Literatur und im deutschen Sprachraum ist das Konzept des MCR trotz seines empirisch gut belegten prognostischen Werts noch kaum bekannt [6, 9, 23, 36].

## Das Konzept des motor-kognitiven Risikosyndroms

### Gang(-geschwindigkeit) und kognitive Leistungsfähigkeit

In der Literatur wird die Ganggeschwindigkeit, die als ein Diagnostikkriterium des MCR genutzt wird, auch als 6. Vitalzeichen bezeichnet, welches eine generelle Einschätzung des allgemeinen Gesundheitsstatus einer Person erlaubt [25].

Eine Abnahme der Ganggeschwindigkeit kann auf viele Ursachen zurückgeführt werden (z. B. Veränderungen des muskuloskelettalen Systems, des kardiopulmonalen Systems oder des sensorischen Systems), welche oftmals in einer komplexen wechselseitigen Beziehung stehen [32]. Folglich ist die über die Altersnorm hinausgehende Abnahme der Ganggeschwindigkeit ein klinisch bedeutsamer, wenn auch wahrscheinlich eher unspezifischer Diagnostikparameter. Dennoch legen zahlreiche Studien den Schluss nahe, dass eine verlangsamte Ganggeschwindigkeit auch einen verlässlichen Indikator und Prädiktor für die Abnahme der kognitiven Leistungsfähigkeit und die Integrität spezifischer Strukturen des zentralen Nervensystems darstellt [19]. Aus klinischer Perspektive ist bedeutsam, dass bereits bis zu 12 Jahre vor einer klinisch erkennbaren Manifestation einer neurodegenerativen Erkrankung (z. B. MCI oder Alzheimer-Demenz) motorische Veränderungen wie die Verlangsamung der Ganggeschwindigkeit beobachtetet werden können [5, 33]. Dementsprechend könnte die Quantifizierung von Ganggeschwindigkeitsveränderungen einen diagnostischen Vorteil bieten, der es erlaubt, frühzeitig Personen mit einem erhöhten Demenzrisiko zu identifizieren [5, 33] und diesen Präventionsmaßnahmen, die beispielsweise auf die Veränderung lebensstilbezogener Verhaltensweisen (z. B. Bewegungsverhalten) abzielen, anzubieten.

## Diagnostik des MCR

**Figure 1:**
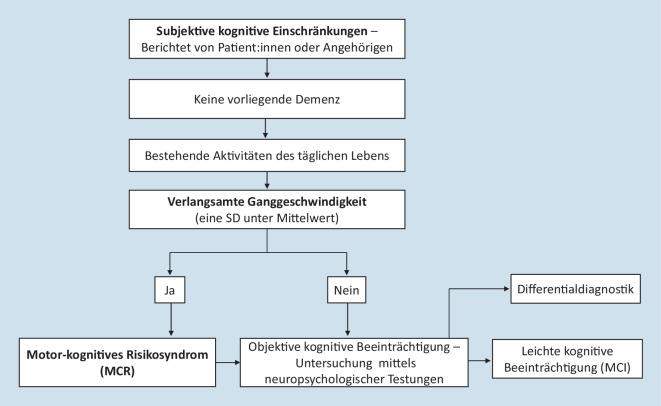

Das MCR wird anhand der vier folgenden Kriterien diagnostiziert [35]. Weiterhin ist in Abb. 1 ein möglicher Diagnosepfad beim Vorliegen einer subjektiv wahrgenommenen kognitiven Einschränkung dargestellt.

1. *Subjektive kognitive Einschränkungen*
  Das Vorliegen einer subjektiv wahrgenommen kognitiven Einschränkung wird durch Selbstauskünfte und spezifische Fragen zum Gedächtnis (z. B. „Haben Sie mehr Probleme mit dem Gedächtnis als andere Menschen?“ und „Ist Ihr Gedächtnis schlechter als vor 10 Jahren?“) ermittelt [36]. In einzelnen Studien wurden auch Angehörige befragt, um weitere Einschätzungen zu erhalten [35].
2. *Verlangsamte Ganggeschwindigkeit*
  Die verlangsamte Ganggeschwindigkeit wurde in den begutachteten Studien mittels einer standardisierten Ganganalyse quantifiziert. Derzeit werden sowohl apparativ unaufwendige klinische Testungen (z. B. der 4‑m-Gang-Test bzw. 10-m-Gang-Test mit Handstoppung) als auch Ganganalysesysteme (z. B. GAITRite-System) zur Bestimmung der Ganggeschwindigkeit verwendet. Als diagnostisch relevant für das MCR gilt eine Ganggeschwindigkeitsabnahme (z. B. in m/s), die eine Standardabweichung unter dem alters- und geschlechtsspezifischen Mittelwert der individuellen Kohorte liegt [35, 36]. In zukünftigen Studien wäre nach Ansicht der Autor:innen dieses Artikels die weitere Standardisierung des Ganggeschwindigkeitsassessments ratsam. Zudem sollte der Faktor der Komorbiditäten (z. B. Schlaganfall, Diabetes oder Arthrose) berücksichtigt werden, um mögliche Einflüsse dieser in die Auswertung der Ganggeschwindigkeit miteinzubeziehen. Inwieweit eine weitere Subtypisierung des motor-kognitiven Risikosyndroms anhand der Variabilität ausgewählter Gangparameter (z. B. Schrittlänge, Schrittbreite) einen Mehrwert bringt, wird derzeitig erforscht [1]. Aufgrund der limitierten Evidenzlage ist aus Sicht der Autor:innen gegenwärtig eine Subtypisierung, anhand ausgewählter Gangparameter, noch nicht von praktischer Relevanz und sollte auch im Hinblick auf die Notwendigkeit einer aufwendigeren Diagnostik (z. B. sensorgestützte Ganganalyse und Auswertung durch Experten) hinsichtlich der klinischen Implementierung des MCR unter der Berücksichtigung von Kosten-Nutzen-Abwägung kritisch betrachtet werden.
3. *Bestehende Aktivitäten des täglichen Lebens*
  Die Aktivitäten des täglichen Lebens können weiter relativ selbstständig ausgeführt werden. Das bedeutet, dass beispielsweise keine Schwierigkeiten beim Anziehen, Essen, Baden, Toilettengang, Kommunizieren und Bewegen bestehen.
4. *Fehlen einer manifesten Demenz*
  Im Rahmen des MCR liegt *keine* Demenzdiagnose vor (ermittelt z. B. nach ICD-10; [35]).

## Prävalenz und Auswirkungen des MCR auf ausgewählte gesundheitsrelevante Parameter

Die Prävalenzrate des MCR wird weltweit zwischen 2 und 27 % angegeben [23], wobei generell ein altersabhängiger Anstieg der Prävalenz beobachtet wurde [22]. Geschlechterunterschiede hinsichtlich der Prävalenzrate konnten nur in vereinzelten Untersuchungen festgestellt werden und die derzeitigen Ergebnisse sind zu inkonsistent, um diesbezüglich reliable Schlussfolgerungen zu ziehen [22, 36]. Im Hinblick auf deutschsprachige Länder liegen derzeitig noch keine Daten zur Prävalenz des MCR vor; hier ist weitere Forschung notwendig.

Eine Reihe von Studien deutet darauf hin, dass Personen mit MCR ein erhöhtes Risiko für das Auftreten negativer gesundheitlicher Ereignisse haben. Beispielsweise wurde in mehreren Studien ein erhöhtes Mortalitäts- [29, 38] und Demenzrisiko bei Personen mit MCR berichtet [23, 29, 32]. In diesem Kontext deuten die Studienergebnisse darauf hin, dass das MCR sowohl mit einem höheren Risiko für das Auftreten einer vaskulären Demenz [5, 35] als auch mit einem erhöhten Risiko für eine Alzheimer-Demenz in Verbindung steht [35, 36]. Die derzeitig noch eher limitierte Studienlage lässt jedoch noch keine zuverlässigen Aussagen zu, inwieweit das Vorliegen des MCR zur Risikostratifizierung spezifischer Formen demenzieller Erkrankungen genutzt werden kann [2], sodass weitere Studien notwendig sind, um die Evidenzlage diesbezüglich zu erweitern.

Neben einem erhöhten Mortalitäts- und Demenzrisiko ist das MCR auch mit dem Konzept der Frailty (multidimensionales geriatrisches Syndrom) und einem erhöhten Sturzrisiko assoziiert [6, 9]. In diesem Zusammenhang wurde in einer großangelegten Beobachtungsstudie mit 6204 Teilnehmer:innen beobachtet, dass Personen mit MCR ein um 44 % erhöhtes Risiko für zukünftige Stürze aufweisen und die Kombination von langsamer Ganggeschwindigkeit und subjektiv wahrgenommener kognitiver Einschränkung ein stärkerer Prädiktor für zukünftige Stürze ist als die einzelnen Prädiktoren alleine [9].

**Figure 2:**
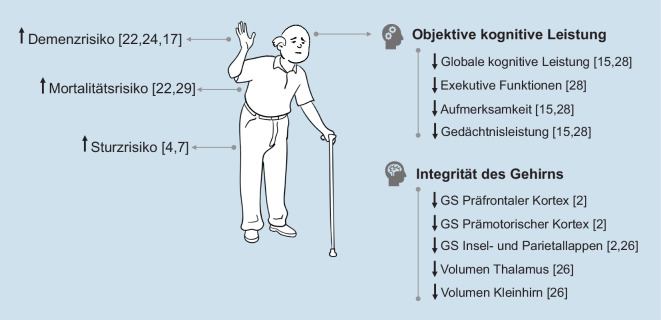

Einen Überblick über Veränderungen gesundheitsbezogener und zentralnervöser Parameter, die mit dem Auftreten des MCR assoziiert sind, bietet Abb. 2. Die in Abb. 2 aufgelisteten Hirnregionen sind u. a. neben der motorischen Kontrolle des Gangs (z. B. präfrontaler Kortex; [16]) auch in verschiedene kognitive Leistungen involviert (z. B. exekutive Funktionen [39]). In der Literatur existieren starke Hinweise, die einen komplexen Zusammenhang zwischen Gehirnveränderungen, kognitiver Leistungsfähigkeit, Gang(-geschwindigkeit) und Sturzrisiko vermuten lassen [16], mögliche Kausalzusammenhänge gilt es im Kontext des MCR jedoch noch näher zu erforschen.

## MCR und MCI – Zusammenhänge und Unterschiede

Das Konzept des MCI und das Konzept des MCR weisen beide eine gewisse Überlappung hinsichtlich ihrer Diagnosekriterien auf, da beide Konzepte darauf abzielen, ein prodromales Stadium demenzieller Erkrankungen zu erfassen. Zugleich handelt es sich beim MCR und MCI um Syndromdiagnosen, Ursachen (z. B. Volumenreduktion des Hippokampus) für das Auftreten müssen zunächst ermittelt und diagnostiziert werden. Wie in Abb. 1 und Tab. 1 dargestellt, ist für beide Konzepte das Vorliegen einer subjektiv wahrgenommenen kognitiven Beeinträchtigung wesentlich, bei der die betroffene Person funktional noch nicht eingeschränkt ist und die Aktivitäten des täglichen Lebens relativ selbständig verrichtet werden können [18, 35]. Die wichtigsten Unterschiede zwischen dem Konzept des MCI und dem Konzept des MCR sind in Tab. 1 dargestellt.

Studien zeigen, dass ca. die Hälfte der Personen, die subjektiv wahrgenommene kognitive Störungen aufweisen, die Kriterien für die Zuordnung zu beiden Konzepten erfüllen, wobei die Literaturangaben bezüglich der Überlappungsrate zwischen 39 und 54 % schwanken [23]. In diesem Kontext ist jedoch beachtenswert, dass der Prädiktionswert des MCR für ein erhöhtes Demenzrisiko auch dann noch statistisch signifikant ist, wenn für das Vorliegen eines MCI adjustiert wurde [35]. Durch die nur partielle Überlappung (im Mittel ca. 50 % der Fälle [35]) werden durch das Konzept des MCR auch Personen, die ein nachweislich höheres Demenzrisiko aufweisen [7, 8, 29], erfasst, welche durch etabliertere Konzepte, wie dem des MCI, nicht identifiziert worden wären [35]. Im Speziellen werden grundsätzlich für die Diagnostik des MCR nur geringe zeitliche, materielle und personelle Ressourcen benötigt, da die Testung der Ganggeschwindigkeit auch von nicht spezifisch geschultem Personal mit vergleichsweise geringem Aufwand (z. B. 4‑m-Gangtest mit Zeitnahme durch Stoppuhr) durchgeführt werden kann und maximal 10 min beansprucht. In diesem Kontext sollte jedoch auch bedacht werden, dass der Diagnostikaufwand durch eine apparative Unterstützung der Ganganalyse ansteigen kann. Da derzeitig unklar ist, inwieweit eine apparativ gestützte Ganganalyse einen relevanten Mehrwert für die MCR-Diagnostik bietet, ist nach Meinung der Autor:innen deren Einsatz durch den größeren Aufwand insbesondere im klinischen Kontext nicht unbedingt erforderlich und gerechtfertigt, wenngleich für Forschungszwecke eine apparativ gestützte Ganganalyse durchaus relevant sein kann. Im Gegensatz zur MCR-Diagnostik erfordert die leitlinienorientierte Diagnostik des MCI sowohl höhere zeitliche Ressourcen als auch besonders geschultes Personal. Beispielsweise werden für die in den S3-Leitlinien Demenz [13] empfohlenen neuropsychologischen Testungen ca. 30–45 min benötigt [31], wobei derzeit auch noch kein international anerkannter Konsensus hinsichtlich der einzusetzenden neuropsychologischen Testverfahren existiert [15].

Insgesamt kann somit festgehalten werden, dass sich die Diagnostik des MCR durch eine höhere Praktikabilität im Vergleich zur Diagnostik des MCI auszeichnet, wenngleich aufgrund der heterogenen und derzeitig limitierten Evidenzlage noch unklar ist, ob der Vorhersagewert des MCR für eine Demenz höher liegt als der des MCI [35, 38]. Die hohe Praktikabilität der MCR-Diagnostik könnte außerdem dazu führen, dass sich das Konzept des MCR auch bei limitierten Ressourcen des Gesundheitssystems (z. B. in Ländern mit niedrigem bis mittleren Einkommen, Landkreisen mit hohem Deprivationsindex) etabliert und somit präventive Maßnahmen früher eingeleitet werden können [29].

## Prävention, Risiko- und Schutzfaktoren des MCR

Für das MCR wird in der Literatur eine Vielzahl nichtmodifizierbarer und modifizierbarer Risikofaktoren genannt, wobei insbesondere die modifizierbaren Risikofaktoren einen Anknüpfungspunkt für gezielte präventive Interventionsmaßnahmen bieten [19].

Die derzeitige Evidenzlage deutet darauf hin, dass insbesondere kardiovaskuläre und metabolische Risikofaktoren die Entstehung des MCR begünstigen [10, 29]. In Anknüpfung an die in Tab. 1 aufgeführten Risikofaktoren, dem Fehlen wirksamer pharmakologischer Therapieansätze [28] und in Anlehnung an generelle Empfehlungen zur Minimierung des Demenzrisikos, scheinen insbesondere multimodale lebensstilbezogene Prä- und Interventionsmaßnahmen [12, 15, 20] vielversprechend zu sein, um dem Auftreten bzw. dem Fortschreiten des MCR entgegenzuwirken. Innerhalb dieser Maßnahmen kommt insbesondere der regelmäßigen körperlichen Aktivität, beispielsweise in Form eines strukturierten körperlichen Trainings, eine übergeordnete Rolle zu [20], da regelmäßige Bewegung mit einem geringeren Risiko für das Auftreten demenzieller Erkrankungen assoziiert ist [12, 20]. Demnach besteht durch eine frühzeitige Diagnostik und Demenzrisikostratifizierung die Möglichkeit, rechtzeitig Interventionen, die auf positive Veränderungen von Lebensstilfaktoren abzielen, einzuleiten, um so dem Abbau der kognitiven Leistungsfähigkeit und neurodegenerativen Veränderungen entgegenwirken zu können. Im Idealfall kann durch die Risikoreduzierung die Autonomie und Lebensqualität der Betroffenen langfristig erhalten werden [20].

Bislang liegt jedoch kaum Evidenz vor, welche Charakteristika für präventive Interventionsmaßnahmen optimal sind, um das Risiko für das Auftreten des MCR und/oder ein weiteres Fortschreiten zu einer demenziellen Erkrankung zu minimieren [23]. In Anlehnung an den „Use-it-or-lose-it“-Ansatz erscheint bei demenziellen Erkrankungen im Allgemeinen und somit möglicherweise auch beim MCR im Speziellen die Durchführung eines kombinierten motor-kognitiven Trainings (z. B. Tanztraining) indiziert, da bei dieser Art des Trainings Übungsformen eingesetzt werden, die körperliche und kognitive Stimulation vereinen. Eigene Studien und Studien anderer Arbeitsgruppen konnten zeigen, dass ein Tanztraining bei gesunden älteren Erwachsenen einen positiven Einfluss auf neurokognitive Parameter [27] und Gangparameter [14] hat. Inwieweit vergleichbare Effekte eines Tanztrainings auch bei Personen mit MCR erreicht werden können, gilt es, durch weitere Forschung zu überprüfen.

## Ausblick

Für die Forschung und die klinische Praxis stellt das MCR ein neues Konzept zur Identifikation von Personen mit erhöhtem Demenzrisiko dar [32], welches es ermöglicht, sowohl die pathophysiologischen Mechanismen demenzieller Erkrankungen besser zu verstehen [32] als auch Personen mit erhöhtem Demenzrisiko frühzeitig präventiven und lebensstilbezogenen Interventionsmaßnahmen zuzuführen [20]. Hierbei ist insbesondere die hohe Praktikabilität der Diagnostik des MCR hervorzuheben, die potenziell auch „remote“ unter Nutzung digitaler Technologien (z. B. zur Erfassung der Ganggeschwindigkeit) erfolgen kann. Neuere Forschungsarbeiten untersuchen derzeit auch den Einsatz von Fragebögen, um ein subjektives MCR zu diagnostizieren [3]. Zudem sollte auch nicht unerwähnt bleiben, dass Konzepte, wie das „mild behavioral impairment“ (MBI), welches durch anhaltende und schwerwiegende neuropsychiatrische Symptome gekennzeichnet und mit einer Reihe von Alzheimer-Biomarkern assoziiert ist, ein weiteres klinische relevantes prodromales Demenzstadion kennzeichnen könnte [11]. Weitere Forschung hinsichtlich möglicher Überlappung verschiedener Konzepte, die auf die Identifizierung prodromaler Demenzstadien abzielen, ist für eine weitere Optimierung der Gesundheitsversorgung der alternden Bevölkerung erforderlich. Insbesondere die Kombination aus verlangsamter Ganggeschwindigkeit und subjektiven kognitiven Beeinträchtigung ist prädikativ für ein erhöhtes Demenzrisiko [29]. Dennoch sollte berücksichtigt werden, dass eine alleinige, über die Altersnorm hinausgehende Ganggeschwindigkeitsreduktion und/oder eine andere Gangstörung nicht imperativ für ein erhöhtes Demenzrisiko sein müssen, sondern ebenso durch andere Einflussfaktoren (z. B. Polyneuropathien, Arthrosen), welche nicht spezifisch mit kognitiven Beschwerden im Zusammenhang stehen, hervorgerufen sein können (z. B. andere neurologische und orthopädische Erkrankungen – siehe für Übersicht [17]). Leser:innen, welche an diesem Thema interessiert sind, verweisen wir auf folgende weiterführende Literatur, die sich vertiefend mit Gangstörungen (z. B. in der Neurologie) und dem Zusammenhang von Gangparametern als Marker zentralnervöser Veränderungen beschäftigt [26, 37]. Obgleich das MCR, gestützt von zahlreichen internationalen Untersuchungen und Analysen, vielversprechende Möglichkeiten für Forschung und Praxis birgt, ist eine weitere empirische Absicherung des Konzepts aus Sicht der Autor:innen notwendig. Insbesondere im deutschsprachigen Raum ist die Datenlage bezüglich etablierter gesundheitswissenschaftlicher Kenngrößen derzeitig limitiert (z. B. Prävalenzrate).

## Fazit für die Praxis

- Charakteristisch für das motor-kognitive Risikosyndrom (MCR) sind subjektive kognitive Einschränkungen sowie eine verlangsamte Ganggeschwindigkeit.
- Personen mit MCR weisen im Vergleich zu gesunden Kontrollpersonen ein erhöhtes Demenz‑, Sturz- und Mortalitätsrisiko auf.
- Die Nutzung der Ganggeschwindigkeit bietet einen praktischen Mehrwert bei der Demenzrisikostratifizierung und kann mit geringen zeitlichen und materiellen Ressourcen durchgeführt werden.
- Präventive bzw. interventionelle Maßnahmen sollten auf modifizierbare Risikofaktoren abzielen – möglich wäre die Durchführung eines motor-kognitiven Trainings.
